# Tetrachloroethylene Exposure and Bladder Cancer Risk: A Meta-Analysis of Dry-Cleaning-Worker Studies

**DOI:** 10.1289/ehp.1307055

**Published:** 2014-03-21

**Authors:** Jelle Vlaanderen, Kurt Straif, Avima Ruder, Aaron Blair, Johnni Hansen, Elsebeth Lynge, Barbara Charbotel, Dana Loomis, Timo Kauppinen, Pentti Kyyronen, Eero Pukkala, Elisabete Weiderpass, Neela Guha

**Affiliations:** 1 Section of Environment and Radiation, and; 2 Section of IARC Monographs, International Agency for Research on Cancer, Lyon, France; 3 National Institute for Occupational Safety and Health, Centers for Disease Control and Prevention, Cincinnati, Ohio, USA; 4 Occupational and Environmental Epidemiology Branch, Division of Cancer Epidemiology and Genetics, National Cancer Institute, National Institutes of Health, Department of Health and Human Services, Bethesda, Maryland, USA; 5 Institute of Cancer Epidemiology, Danish Cancer Society, Copenhagen, Denmark; 6 Department of Public Health, University of Copenhagen, Copenhagen, Denmark; 7 Université de Lyon/Unité Mixte de Recherche Epidémiologique et de Surveillance Transport Travail Environnement (IFSTTAR/UCBL), Lyon, France; 8 Finnish Institute of Occupational Health, Helsinki, Finland; 9 Finnish Cancer Registry, Institute for Statistical and Epidemiological Cancer Research, Helsinki, Finland; 10 School of Health Sciences, University of Tampere, Tampere, Finland; 11 Cancer Registry of Norway, Oslo, Norway; 12 Department of Medical Epidemiology and Biostatistics, Karolinska Institutet, Stockholm, Sweden; 13 Department of Community Medicine, Faculty of Health Sciences, University of Tromsø, The Arctic University of Norway, Tromsø, Norway; 14 Folkhälsan Research Center, Samfundet Folkhälsan, Helsinki, Finland

## Abstract

Background: In 2012, the International Agency for Research on Cancer classified tetrachloroethylene, used in the production of chemicals and the primary solvent used in dry cleaning, as “probably carcinogenic to humans” based on limited evidence of an increased risk of bladder cancer in dry cleaners.

Objectives: We assessed the epidemiological evidence for the association between tetrachloroethylene exposure and bladder cancer from published studies estimating occupational exposure to tetrachloroethylene or in workers in the dry-cleaning industry.

Methods: Random-effects meta-analyses were carried out separately for occupational exposure to tetrachloroethylene and employment as a dry cleaner. We qualitatively summarized exposure–response data because of the limited number of studies available.

Results: The meta-relative risk (mRR) among tetrachloroethylene-exposed workers was 1.08 (95% CI: 0.82, 1.42; three studies; 463 exposed cases). For employment as a dry cleaner, the overall mRR was 1.47 (95% CI: 1.16, 1.85; seven studies; 139 exposed cases), and for smoking-adjusted studies, the mRR was 1.50 (95% CI: 0.80, 2.84; 4 case–control studies).

Conclusions: Our meta-analysis demonstrates an increased risk of bladder cancer in dry cleaners, reported in both cohort and case–control studies, and some evidence for an exposure–response relationship. Although dry cleaners incur mixed exposures, tetrachloroethylene could be responsible for the excess risk of bladder cancer because it is the primary solvent used and it is the only chemical commonly used by dry cleaners that is currently identified as a potential bladder carcinogen. Relatively crude approaches in exposure assessment in the studies of “tetrachloroethylene-exposed workers” may have attenuated the relative risks.

Citation: Vlaanderen J, Straif K, Ruder A, Blair A, Hansen J, Lynge E, Charbotel B, Loomis D, Kauppinen T, Kyyronen P, Pukkala E, Weiderpass E, Guha N. 2014. Tetrachloroethylene exposure and bladder cancer risk: a meta-analysis of dry-cleaning-worker studies. Environ Health Perspect 122:661–666; http://dx.doi.org/10.1289/ehp.1307055

## Introduction

Bladder cancer is the ninth most common cancer diagnosis worldwide, with > 330,000 estimated new cases and > 130,000 estimated deaths each year (Ferlay et al. 2010). Although cigarette smoking is the most important risk factor for bladder cancer, accounting for approximately 66% of new cases in men and 30% of the cases in women in industrialized populations (Burger et al. 2013), an increased risk of bladder cancer has also been reported among persons employed in certain industries (e.g., rubber production, aluminum production, textile and dye manufacturing) and occupations (e.g., painter, hair dresser/barber, dry cleaners) [Guha et al. 2010; International Agency for Research on Cancer (IARC) 2009b], and in relation to exposure to specific chemicals (e.g., aromatic amines, polycyclic aromatic hydrocarbons, arsenic, tetrachloroethylene) [Guha et al. 2012; IARC 2009a; U.S. Environmental Protction Agency (EPA) 2012].

Tetrachloroethylene (also referred to as perchloroethylene) is one of the most important chlorinated solvents worldwide and has been produced commercially since the early 1900s. Currently the primary use of tetrachloroethylene is as a raw material for the production of fluorocarbons (Guha et al. 2012). However, between the 1950s and 1980s, most of the tetrachloroethylene that was produced was used in dry cleaning (Doherty 2000), with smaller amounts used for degreasing metals and in the production of chlorofluorocarbons.

Epidemiological studies of workers provide a good platform for identifying individuals with considerable exposure to tetrachloroethylene. To date, few epidemiological studies assessing bladder cancer risk have included quantitative estimates of occupational exposure to tetrachloroethylene. However, some insight into the relationship between bladder cancer risk and exposure to tetrachloroethylene can be gained by studies of workers in the dry-cleaning industry.

CAREX, an international, country-specific survey of occupational exposure to carcinogens, reported that the majority of the workers occupationally exposed to tetrachloroethylene were employed in dry-cleaning shops (Kauppinen et al. 2000). The prevalence of exposure among dry cleaners was reported at 70% in the United States in 2007 (Halogenated Solvents Industry Alliance 2008), and 90% in France and two-thirds in Denmark in 2012 (European Chlorinated Solvent Association 2013). Although limited quantitative exposure data are available, some dry cleaners may have been heavily exposed to tetrachloroethylene. Before the 1960s, most dry cleaners manually moved garments immersed in tetrachloroethylene from washers to dryers—a practice that may still exist today among those using older equipment (Garetano and Gochfeld 2000)—that may result in high dermal exposure.

Epidemiological findings of an increased risk of bladder cancer in dry cleaners exposed to tetrachloroethylene led an expert working group assembled by the Monographs Programme at IARC to reaffirm the classification of tetrachloroethylene as “probably carcinogenic to humans” (Group 2A) in October 2012 and to newly identify the bladder as a target organ (Guha et al. 2012). For this assessment, the working group carefully reviewed the data on human exposure, carcinogenesis bioassays in experimental animals, and the mechanisms of carcinogenesis, in addition to the epidemiological findings of cancer in humans (Guha et al. 2012). There were no mechanistic data to inform the increased risk of bladder cancer in people exposed to tetrachloroethylene. The working group did identify several potential genotoxic and nongenotoxic mechanisms of carcinogenesis for tetrachloroethylene in the liver from cancer bioassays in mice and toxicity studies in rodents that could operate in humans. In rats, tetrachloroethylene has been shown to induce neoplasms of the hematopoietic system, testes, kidney, and brain, although the human cancer data were not as strong for these sites (Guha et al. 2012; U.S. EPA 2012).

To complement the systematic IARC review, we conducted meta-analyses of published studies that specifically assessed occupational exposure to tetrachloroethylene or studies of dry-cleaning workers to further evaluate evidence for the risk of bladder cancer associated with tetrachloroethylene exposure. We qualitatively assessed exposure–response relationships from the limited number of studies available.

## Methods

We conducted a literature search for publications in any language that reported risk estimates for bladder cancer in relation to occupational exposure to tetrachloroethylene or provided enough information for their calculation. We identified studies from the 2012 IARC evaluation of the carcinogenicity of tetrachloroethylene (Guha et al. 2012) and the U.S. EPA review of tetrachloroethylene (U.S. EPA 2012). In addition, we searched PubMed (http://www.ncbi.nlm.nih.gov/pubmed) using the following keywords: “dry cleaners,” “dry cleaning,” “occupation,” “tetrachloroethylene,” “bladder cancer,” “bladder carcinoma,” and “urothelial carcinoma” in various combinations. Searches using common variations on these keywords did not result in the identification of additional studies.

We included studies that reported a risk estimate specifically for “tetrachloroethylene-exposed workers” or for employment as a “dry cleaner,” because of historical information indicating that many dry cleaners were exposed to tetrachloroethylene but generally not to other known or suspected occupational bladder carcinogens (IARC 1995). We included risk estimates that were reported for men and women combined. If a study reported risk estimates for men and women separately, we included both risk estimates separately in the meta-analyses. If a study reported results stratified by exposure groups and not for “any occupational exposure” versus “background exposure,” we pooled the risk estimates by conducting a within-study random-effects meta-analysis of the nonreference exposure groups. Several studies reported results only for the occupational category “dry-cleaning and laundry workers.” We conducted a sensitivity analysis with the expectation that laundry workers were unexposed to tetrachloroethylene or were exposed only at background levels; therefore, risk estimates would be biased toward the null for a combined occupational category of dry-cleaning and laundry workers because of unexposed or lightly exposed individuals misclassified as exposed.

We excluded studies that reported proportional mortality analyses because the risk estimates are potentially biased. When several publications were available from a single study population, we considered only the most complete or recent publication. Four overlapping papers in the U.S. National Cancer Institute (NCI) National Bladder Cancer Study reported findings for bladder cancer risk in dry cleaners and/or launderers (Schoenberg et al. 1984; Silverman et al. 1989, 1990; Smith et al. 1985). Of these, only two (Silverman et al. 1989, 1990) were included in the sensitivity analysis for laundry and dry-cleaning workers because of the significant, but not clearly specified, overlap between the study populations and because of information indicating that laundry and dry-cleaning workers were combined by Schoenberg et al. (1984), which was not stated in the article (Silverman D, personal communication). Publications included in the meta-analysis are listed in Table 1.

**Table 1:** Overview of publications included in the meta-analysis.

Study ID	Reference	Country	Study design	Sex	Disease classification	Exposure definition	Exposure perioda	Smoking adjusted^*b*^	Exposed cases (*n*)	I or M	Risk estimate
1	Blair et al. 2003	USA	Cohort	Both	188 (ICDA-8)^*c*^	Dry cleaning	< 1979d	no	12	M	SMR
2	Calvert et al. 2011	USA	Cohort	Both	188, 189.3–189.9 (ICD-9)	Dry cleaning	< 1982e	no	10	M	SMR
3	Lipworth et al. 2011	USA	Cohort	Both	188, 189.3–189.9 (ICD-9)	Tetrachloroethylene	< 1996d	no	17	M	SMR
4	Lynge et al. 2006f	D,N,S,Fg	Cohort	Both	C67 (ICD-O2)^*h*^	Dry cleaning	< 1970	no	93	I	RR
4	Pukkala et al. 2009f	D,N,S,Fg	Cohort	Both	181 (ICD-7)	Laundry or dry cleaning	< 1970	no	186	I	SIR
5	Burns and Swanson 1991	USA	Case–control	Both	Not reported	Dry cleaning	< 1991i	yes	8	I	OR
6	Siemiatycki 1991	Canada	Case–control	Men	188 (ICD-9)	Laundry or dry cleaning	< 1985	yes	10	I	OR
6	Christensen et al. 2013j	Canada	Case–control	Men	188 (ICD-9)	Tetrachloroethylene	< 1985	yes	2	I	OR
7	Colt et al. 2011	USA	Case–control	Men	k	Dry cleaning	< 2004	yes	4	I	OR
7	Colt et al. 2011	USA	Case–control	Women	k	Dry cleaning	< 2004	yes	6	I	OR
8	Dryson et al. 2008	New Zealand	Case–control	Both	Not reported	Laundry or dry cleaning	< 2004	yes	3	I	OR
9	Gaertner et al. 2004	Canada	Case–control	Men	l	Dry cleaning	< 1997	yes	4	I	OR
10	Kogevinas et al. 2003	Western Europem	Case–control	Men	Not reported	Laundry or dry cleaning	< 1995	yes	19	I	OR
11	Pesch et al. 2000	Germany	Case–control	Both	n	Tetrachloroethylene	< 1995	yes	444	I	OR
12	Silverman et al. 1989	USA	Case–control	Men (nonwhite)	l,o	Laundry or dry cleaning	< 1978	yes	11	I	OR
12	Silverman et al. 1990	USA	Case–control	Women	l,o	Laundry or dry cleaning	< 1978	yes	23	I	OR
13	Steineck et al. 1990	Sweden	Case–control	Men	o	Dry cleaning	< 1987	yes	2	I	OR^*p*^^**^
14	Teschke et al. 1997	Canada	Case–control	Both	188 (ICD-O)	Laundry or dry cleaning	< 1991	yes	5	I	OR
15	Zheng et al. 2002	USA	Case–control	Women	l	Laundry or dry cleaning	< 1989	yes	3	I	OR
Abbreviations, I, incidence; ICD, *International Classification of Diseases*; M, mortality; OR, odds ratio; RR, rate ratio; SIR, standardized incidence ratio; SMR, standardized mortality ratio. ^***a***^Assumed date of last exposure, based on last reported date of case inclusion; exposures prior to 1960 could have included other solvents, such as carbon tetra­chloride or Stoddard solvent. ^***b***^Included relative risk, smoking-adjusted (yes/no). ^***c***^ICD-8 adapted for use in the United States. ^***d***^Earliest date of entry into cohort was 1948. ^***e***^Mean year first employed was 1953; monitoring data were used to exclude workers who had been exposed to carbon tetrachloride or trichloroethylene. ^***f***^There is considerable overlap between the cohort used for Pukkala et al. (2009) and the cohort used for Lynge et al. (2006); therefore, the risk estimates are not combined in the meta-analysis. Pukkala et al. (2009) reported results for laundry or dry-cleaning workers, whereas Lynge et al. (2006) reported results for dry-cleaning workers only. Accordingly, the studies were included in the respective meta-analyses. ^***g***^Denmark, Norway, Sweden, and Finland. ^***h***^*International Classification of Diseases for Oncology*, 2nd ed. ^***i***^Based on date of publication; no case inclusion dates were reported. ^***j***^Results based on population controls are included (results based on hospital controls also reported). ^***k***^Histologically confirmed carcinoma of the urinary bladder (including carcinoma *in situ*). ^***l***^Histologically confirmed bladder cancer. ^***m***^Denmark, France, Germany, Italy, Spain. ^***n***^Histologically confirmed cancer of the urinary bladder, ureter, and renal pelvis. ^***o***^Urothelial cancer and/or squamous-cell carcinoma in the lower urinary tract. ^***p***^Results from conditional logistic regression.											

We conducted random-effects meta-analyses to pool the relative risks (RRs) reported in the included publications (Table 2). We analyzed separately the studies reporting on tetrachloroethylene-exposed workers and the studies reporting on dry-cleaning workers. We used an α of 0.05 to assess whether meta-relative risks (mRRs) were significantly elevated. Inconsistency among the studies was quantified using the *I*^2^ statistic (Higgins et al. 2003). *I*^2^ values of 25–50% indicate moderate inconsistency, whereas values > 50% reflect large inconsistencies among studies. We assessed the sensitivity of the outcome of the meta-analyses by excluding individual studies one at a time and also by restricting the analyses to certain subgroups (i.e., studies reporting a RR for “employment as dry cleaner,” cohort studies, case–control studies, studies that adjusted for smoking). We assessed publication bias visually through a funnel plot and quantitatively with Egger’s graphical test (evidence of publication bias if the Egger’s test *p*-value was < 0.05) (Egger et al. 1997). We compared mRRs by strata using a test of interaction (Altman and Bland 2003).

**Table 2:** Meta-analysis of studies reporting exposure to tetrachloroethylene or employment in dry cleaning and the risk of bladder cancer.

Study base	No. of**studies	Exposed cases (*n*)	mRR (95% CI)	*I *^2^ (%)	ID of studies includeda
Tetrachloroethylene-exposed workers
With Pesch et al. 2000 JEM results	3	463	1.08 (0.82, 1.42)	25.3	3, 6, 11
With Pesch et al. 2000 JTEM results	3	125	1.05 (0.76, 1.47)	19.6	3, 6, 11
Laundry and dry-cleaning workers	13	306	1.20 (1.06, 1.36)	0.0	1, 2, 4, 5, 6, 7, 8, 9, 10, 12, 13, 14, 15
Cohort studies^*b*^	3	208	1.17 (0.95, 1.44)	13.1	1, 2, 4
Case–control studies^*c*^	11	98	1.54 (1.17, 2.04)	0.0	4, 5, 6, 7, 8, 9, 10, 12, 13, 14, 15
Dry-cleaning workers	7	139	1.47 (1.16, 1.85)	0.0	1, 2, 4, 5, 7, 9, 13
Excluding Lynge et al. 2006	6	46	1.51 (1.05, 2.18)	0.0	1, 2, 5, 7, 9, 13
Cohort studies^*b*^	3	115	1.46 (1.14, 1.87)	0.0	1, 2, 4
Case–control studies^*c*^	4	24	1.50 (0.80, 2.84)	0.0	5, 7, 9, 13
Abbreviations: JEM, job exposure matrix; JTEM, job-task exposure matrix; mRR, meta-relative risk. ^***a***^Study IDs are given in Table 1. ^***b***^None of the cohort studies was directly adjusted for smoking behavior. ^***c***^All case–control analyses were adjusted for smoking behavior.					

We qualitatively summarized the exposure–response data (e.g., duration of employment as a dry cleaner or duration or intensity of exposure to tetrachloroethylene) because of the limited number of studies available (Table 3). We conducted all statistical analyses in Stata (version 11; StataCorp LP, College Station, TX, USA).

**Table 3:** Exposure–response information available in studies included in the meta-analysis.

Study and exposure	Association	No. of cases
Pesch et al. 2000; tetrachloroethylene exposure index^*a*^
Men^*b*^
Medium	OR = 1.1 (0.9, 1.3)	162
High	OR = 1.2 (1.0, 1.5)	172
Substantial	OR = 1.4 (1.0, 1.9)	71
Men^*c*^
Medium	OR = 1.0 (0.7, 1.5)	37
High	OR = 1.2 (0.8, 1.7)	47
Substantial	OR = 1.8 (1.1, 3.1)	22
Women^*b*^
Medium	OR = 1.8 (1.0, 3.0)	21
High	OR = 1.0 (0.6, 1.9)	16
Substantial	OR = 0.7 (0.2, 2.5)	3
Christensen et al. 2013; tetrachloroethylene exposure
Any exposure	OR = 0.5 (0.1, 3.0)	2
Substantial exposure^*d*^	OR = 0.9 (0.1, 7.3)	2
Blair et al. 2003; duration in the union
< 4.4 years	SMR = 1.4	Not reported
> 4.4 years	SMR = 1.5	Not reported
Blair et al. 2003; level of exposure to dry-cleaning solvents
Little/no	SMR = 1.4 (0.4, 3.2)	5
Medium/high	SMR = 1.5 (0.6, 3.1)	7
Lynge et al. 2006; duration of employment as dry cleaner (years)
0–1^*e*^	RR = 1.50 (0.57, 3.96)	6
2–4	RR = 2.39 (1.09, 5.22)	10
5–9	RR = 0.91 (0.52, 1.59)	17
≥ 10	RR = 1.57 (1.07, 2.29)	53
Calvert et al. 2011; duration of exposure among workers for which time since exposure was > 20 years^*f*^
< 5 years	SMR = 0.53 (0.03, 2.52)	1
> 5 years	SMR = 4.08 (2.13, 7.12)	9
Abbreviations: OR, odds ratio; RR, rate ratio; SMR, standardized mortality ratio. Values in parentheses are 95% CIs.^***a***^Product of duration, probability, and intensity of exposure to tetrachloroethylene. ^***b***^Based on job exposure matrix (JEM) estimates. ^***c***^Based on job-task exposure matrix (JTEM) estimates. ^***d***^To be classified as exposed at the substantial level, a subject had to have been exposed at a confidence level of probable or definite, at a concentration and frequency of medium or high, and for a duration > 5 years. ^***e***^Lynge et al. (2006) defined the lowest exposure category as 0–1 year; however, because the exposed cases and controls were categorized only by length of employment in the shop where they worked in 1970, we changed the lower bound of this category to > 0 for our meta-analysis. ^***f***^No bladder cancer deaths were observed among any of the workers with time since exposure < 20 years.		

## Results

We identified 38 publications from 26 studies that assessed the risk of bladder cancer among tetrachloroethylene-exposed workers or among dry-cleaning workers (13 case–control studies, 11 cohort studies, 1 meta-analysis, and 1 cluster analysis). We excluded 20 publications from the meta-analyses because *a*) they reported standardized mortality odds ratios (1 study) or proportionate mortality ratios (4 studies); *b*) the extent of exposure to tetrachloroethylene was unclear (4 studies); *c*) the publication was superseded by a more recent publication (1 study); *d*) the study population overlapped that of another publication (9 studies); or *e*) the publication was a meta-analysis (1 study). An overview of these publications and the rationale for excluding them from our meta-analysis is provided in Supplemental Material, Table S1. Table 1 presents more details of the studies that were included in the present meta-analyses.

*Tetrachloroethylene-exposed workers*. We included one cohort study (Lipworth et al. 2011) and two case–control studies (Christensen et al. 2013; Pesch et al. 2000) that assessed the risk of bladder cancer among tetrachloroethylene-exposed workers (Table 1). Risk estimates were adjusted for smoking in both case–control studies but not in the cohort study. With the exception of one study that reported results for urothelial cancer (Pesch et al. 2000), all studies reported results for all bladder cancer subtypes combined.

To allow inclusion into this meta-analysis, we had to pool multiple nonreference exposure group–specific odds ratios (ORs) for the Pesch et al. (2000) study, which reported results based on a job exposure matrix (JEM) and also on a (more precise) job-task exposure matrix (JTEM). Because the JEM results were based on a much larger number of cases than were the JTEM results (445 vs. 106), we included these in the meta-analysis and we assessed the sensitivity of the mRR for this decision. The overall mRR for bladder cancer in studies of tetrachloroethylene-exposed workers was 1.08 (95% CI: 0.82, 1.42) (Table 2). When we substituted the JEM-based results from Pesch et al. (2000) (OR = 1.19; 95% CI: 1.06, 1.34) with the JTEM-based results (OR = 1.24; 95% CI: 0.91, 1.69), the mRR was 1.05 (95% CI: 0.76, 1.47) (Table 2). For the studies included in our meta-analysis, we found no evidence of between-study heterogeneity (*I*^2^ < 30%) or publication bias (Egger’s test *p*-value, > 0.05). Considering the limited number of studies available, we did not conduct a separate meta-analysis on the two available case–control studies.

*Dry-cleaning worker studies*. We included 3 cohort studies (Blair et al. 2003; Calvert et al. 2011; Pukkala et al. 2009) and 11 case–control studies (Burns and Swanson 1991; Colt et al. 2011; Dryson et al. 2008; Gaertner et al. 2004; Kogevinas et al. 2003; Siemiatycki 1991; Silverman et al. 1989, 1990; Smith et al. 1985; Steineck et al. 1990; Teschke et al. 1997; Zheng et al. 2002) that assessed the risk of bladder cancer among dry-cleaning workers, or dry-cleaning and laundry workers (Table 1).

The overall mRR for bladder cancer in studies with laundry and/or dry-cleaning workers was 1.20 (95% CI: 1.06, 1.36). The mRR was 1.17 (95% CI: 0.95, 1.44) among cohort studies and 1.54 (95% CI: 1.17, 2.04) among case–control studies (Table 2). One study reported results for urothelial cancer (Steineck et al. 1990), and the other studies reported results for all bladder cancer subtypes combined. We did not observe evidence for between-study heterogeneity (*I*^2^ < 30%). Some evidence for publication bias was observed in this meta-analysis using Egger’s test (*p*-value, 0.013).

**Figure 1:**
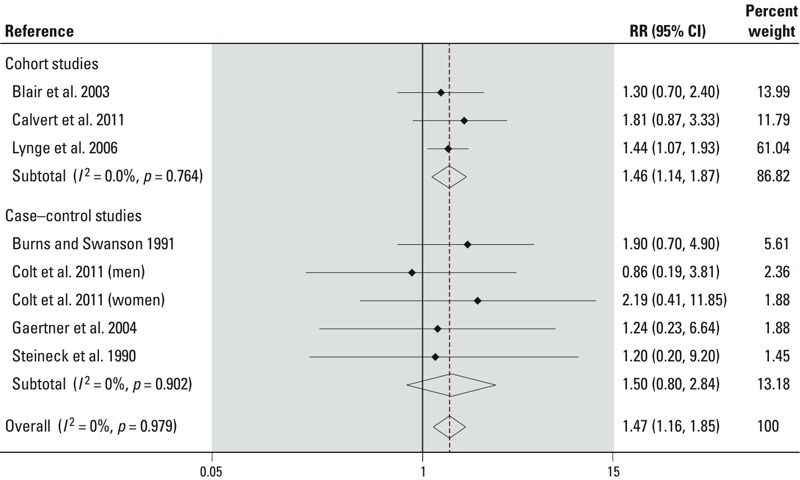
Forest plot of cohort and case–control studies included in the meta-analysis that assessed the risk of bladder cancer in relation to occupation as a dry cleaner. See Table 1 for details on included studies.

We included eight risk estimates from seven studies that assessed the risk of bladder cancer among dry-cleaning workers only (Blair et al. 2003; Burns and Swanson 1991; Calvert et al. 2011; Colt et al. 2011; Gaertner et al. 2004; Lynge et al. 2006; Steineck et al. 1990) (Table 1, Figure 1). One publication reported sex-specific risk estimates (Colt et al. 2011), which we included for men and women separately. We included Lynge et al. (2006) instead of Pukkala et al. (2009) because of the considerable overlap between the cohorts studied in these publications. Lynge et al. (2006) reported results for dry-cleaning workers only, whereas Pukkala et al. (2009) reported results for the combined category of laundry or dry-cleaning workers.

The overall mRR for bladder cancer in studies of dry-cleaning workers was 1.47 (95% CI: 1.16, 1.85). The mRR was 1.46 (95% CI: 1.14, 1.87) among cohort studies and 1.50 (95% CI: 0.80, 2.84) among case–control studies (Table 2, Figure 1). In all case–control studies included in this analysis, risk estimates were adjusted for smoking. Although the cohort studies did not adjust for smoking, one study used unexposed laundry workers as the comparison group in order to indirectly control for tobacco use because the smoking pattern in those two groups are expected to be similar (Lynge et al. 2006). We did not observe evidence for between-study heterogeneity (*I*^2^ < 30%) or publication bias (Egger’s test *p-*value, > 0.05) in this meta-analysis. Although one study had considerable weight (60.6%) (Lynge et al. 2006), excluding it did not have a considerable impact on the mRR (1.51; 95% CI: 1.05, 2.18; six studies) (Table 2).

*Exposure–response information reported in the published studies*. Five studies included in the meta-analyses provided information on the exposure–response relationship based on duration or intensity of exposure to tetrachloroethylene (two studies) or duration of employment as a dry cleaner (three studies) and bladder cancer risk (Blair et al. 2003; Calvert et al. 2011; Christensen et al. 2013; Lynge et al. 2006; Pesch et al. 2000). Exposure group–specific risk estimates for these studies are reported in Table 3. In general, we observed some evidence of an exposure–response association in these five studies.

Only Pesch et al. (2000) provided some evidence for an upward trend in ORs with increasing exposure index (product of duration, probability, and intensity of exposure to tetrachloroethylene). For men, ORs based on the JTEM exposure assessment increased with exposure index: 1.0 (95% CI: 0.7, 1.5; *n* = 37 cases) for medium exposure (> 30th percentile of the distribution of exposure among exposed controls), 1.2 (95% CI: 0.8, 1.7; *n* = 47 cases) for high exposure (> 60th percentile of the distribution of exposure among exposed controls), and 1.8 (95% 1.1, 3.1; *n* = 22 cases) for substantial exposure (> 90th percentile of the distribution of exposure among exposed controls). ORs based on the JEM exposure assessment (405 exposed cases) also increased with increasing exposure index, although they were less pronounced. For women (40 exposed cases), results based only on the JEM exposure assessment were reported, and no upward trend was observed. Lynge et al. (2006) reported RRs by duration of exposure. RRs were 1.50 (95% CI: 0.57, 3.96) for workers exposed for < 1 year, 2.39 (95% CI: 1.09, 5.22) for those exposed 2–4 years, 0.91 (95% CI: 0.52, 1.59) for those exposed 5–9 years, and 1.57 (95% CI: 1.07, 2.29) for those exposed ≥ 10 years. In the remaining studies (Blair et al. 2003; Calvert et al. 2011; Christensen et al. 2013) assessment of the exposure–response relationship was impaired by the limited number of cases.

## Discussion

In our meta-analysis we assessed studies of dry-cleaning (and laundry) workers to gain insight into the potential association between exposure to tetrachloroethylene and bladder cancer risk. Ideally, the highest quality evidence to assess this association would come from studies that conducted quantitative assessment of exposure to tetrachloroethylene (Vlaanderen et al. 2008). However, we identified only three studies that estimated exposure to tetrachloroethylene specifically (Christensen et al. 2013; Lipworth et al. 2011; Pesch et al. 2000), none of which reported estimates of risk per unit of exposure to tetrachloroethylene. These studies used relatively crude methods to generate exposure estimates (i.e., using only job-title information to assign exposure), which would likely result in considerable nondifferential misclassification of exposure, thereby biasing the risk estimates towards the null (Blair et al. 2007).

Several different approaches were used to classify individuals into occupational categories in studies of dry cleaners. Because of the large number of small shops and the high turnover in this industry, two studies assembled cohorts through union records (Blair et al. 2003; Calvert et al. 2011). In these studies information was available only on job title at entry into the cohort (i.e., data at entry into the union). Both studies augmented job-title information with monitoring data. Blair et al. (2003) used monitoring data from other studies of the dry-cleaning industry to assign an exposure score to the jobs held. Calvert et al. (2011) used monitoring data to verify exposure to tetrachloroethylene and other dry-cleaning solvents, and to exclude workers who had been exposed to carbon tetrachloride or trichloroethylene. A similar approach was used by Lynge et al. (2006), who supplemented census and registry data with implied exposure status (working as a dry cleaner or in a dry-cleaning shop) on the basis of original information from the census forms (Denmark and Norway), interviews (Sweden), and pension scheme data (Finland). In the case–control studies (Burns and Swanson 1991; Colt et al. 2011; Gaertner et al. 2004; Steineck et al. 1990), classification into occupational categories was based on information from interviewers. Available information—including a full occupational history, complete description of the duties performed, and the dates each job began and ended—was categorized using occupational classification standards.

Differences in exposure assessment strategies reflect the design of the studies. Although information on the full working history would be preferred over a “snapshot” of an individual’s job title at a specific point in time, acquiring such information is often difficult in large cohort studies.

Our finding of a lower mRR in studies that combined laundry and dry-cleaning workers than among studies including only dry-cleaning workers supports our hypothesis that laundry workers may have received little or no exposure to tetrachloroethylene. A possible explanation for the higher mRR among the dry-cleaning worker studies than among the tetrachloroethylene-exposed worker studies would be co-exposure to a yet unidentified occupational bladder carcinogen, although there are no clear candidates. It is also possible that dry-cleaning workers have lifestyle factors that could account for the observed excess. Blair et al. (2003) observed higher bladder cancer mortality in dry cleaners after the introduction of tetrachloroethylene, supporting the hypothesis that tetrachloroethylene may in fact be responsible for the cancer excess. Further, relatively crude exposure assessment approaches in the studies of tetrachloroethylene-exposed workers might have attenuated the relative risks. Finally, the differences in the mRRs between these groups are not large and may just be due to chance occurrences (*p*-value for interaction = 0.11).

Smoking is the most important risk factor for bladder cancer and accounts for approximately one-half of all cases (Burger et al. 2013). None of the cohort studies included in our meta-analysis specifically controlled for tobacco smoking, although Lynge et al. (2006) used unexposed laundry workers as the comparison group as an indirect proxy for bladder cancer risk factors such as tobacco use. The assumption is that the socioeconomic status of launderers and dry cleaners is similar, which should provide some control for socioeconomic status–related factors. Among the subgroup of dry-cleaning workers only, the mRR for the case–control studies that adjusted for tobacco smoking was similar to the mRR for the cohort studies, indicating that there is little evidence of confounding by tobacco smoking. One case–control study (Colt et al. 2011) assessed and reported no interaction between the OR for tobacco smoking and the OR for dry-cleaning workers.

Finally, it is important to note that although dry cleaners were exposed to other chemicals, they were primarily exposed to tetrachloroethylene. Before 1960, dry-cleaning workers could also have been exposed to carbon tetrachloride or Stoddard solvent (IARC 1995), although these chemicals have not been classified as bladder carcinogens by IARC. [IARC did classify carbon tetrachloride as “possibly carcinogenic to humans” based on excess liver and mammary neoplasms in experimental animals exposed to carbon tetrachloride (IARC 1999).] Although occupational exposure to aromatic amines, arsenic, and possibly polycyclic aromatic hydrocarbons are other risk factors for bladder cancer (IARC 2009a, 2009b), these exposures are unlikely to be confounders because dry-cleaning workers are generally not occupationally exposed to these agents. However, it is possible that exposure to these agents may have occurred during jobs held before or after employment as a dry-cleaning worker.

Our finding of an increase in bladder cancer risk among dry-cleaning workers is consistent with two other reviews. In a meta-analysis of 14 studies of dry cleaners and launderers (our meta-analysis includes 13 studies), Reulen et al. (2008) reported an mRR of 1.27 (95% CI: 0.95, 1.71). A recent systematic literature review by the U.S. EPA also concluded that bladder cancer was one of the human tumor types associated with tetrachloroethylene exposure. The U.S. EPA characterized tetrachloroethylene as “likely to be carcinogenic to humans” based on suggestive evidence of carcinogenicity in epidemiological studies and conclusive evidence of tumorigenicity in rodents (U.S. EPA 2012).

## Conclusion

In a meta-analysis of seven studies of dry-cleaning workers, we observed a significantly elevated risk of bladder cancer. This excess occurred in both cohort and case–control studies. The outcome of our meta-analysis was not excessively sensitive to individual studies or study types. Among studies with the necessary information, the excesses did not appear to be confounded by smoking behavior. In the few studies that provided information on exposure–response (e.g., duration of employment as a dry cleaner or duration or intensity of exposure to tetrachloroethylene), we observed no clear patterns. Our results demonstrate that workers in the dry-cleaning industry experienced an elevated risk of bladder cancer. Dry cleaners were exposed to a mixture of solvents, with tetrachloroethylene being the only component of the mixture identified as a potential bladder carcinogen. Therefore, the higher risk of bladder cancer in dry cleaners may have been due to tetrachloroethylene exposure, the primary solvent used in dry cleaning. However, with limited evidence from studies that specifically assessed exposure to tetrachloroethylene, we were not able to corroborate this hypothesis.

## Supplemental Material

(187 KB) PDF
