# Supplementary material for: Tetrachloroethylene Exposure and Bladder Cancer Risk: A Meta-Analysis of Dry-Cleaning-Worker Studies
Source: Environ Health Perspect. 2014 Mar 21;122(7):661–6. doi: 10.1289/ehp.1307055 (PMC4080536; doi:10.1289/ehp.1307055)
Supplement: (187 KB) PDF [file ehp.1307055.s001.pdf]

Environmental Health Perspectives doi:10.1289/ehp.1307055

Supplemental Material

Tetrachloroethylene Exposure and Bladder Cancer Risk: A Meta-Analysis of Dry-Cleaning-Worker Studies

Jelle Vlaanderen, Kurt Straif, Avima Ruder, Aaron Blair, Johnni Hansen, Elsebeth Lynge, Barbara Charbotel, Dana Loomis, Timo Kauppinen, Pentti Kyyronen, Eero Pukkala, Elisabete Weiderpass, and Neela Guha

Table S1. Overview of studies excluded from the meta-analysis.

Sources	Study design	Exposure category	Comments
Andersen et al. 1999	cohort	laundry and dry cleaning workers	study population overlaps with (Pukkala et al. 2009), which includes a larger number of individuals
Aschengrau et al. 1993	case-control	exposure through contaminated drinking water	extent exposure to tetrachloroethylene is unclear
Boice et al. 1999	cohort	exposure to tetrachloroethylene	superseded by (Lipworth et al. 2011)
Chang et al. 2005	cohort	workers employed in an electronics factory	extent exposure to tetrachloroethylene is unclear
Duh and Asal 1984	cohort	laundry and dry cleaning workers	reports standardized mortality odds ratios
Ji et al. 2005	cohort	laundry and dry cleaning workers	study population overlaps with (Pukkala et al. 2009), which includes a larger number of individuals
Katz and Jowett 1981	cohort	laundry and dry cleaning workers	reports proportionate mortality ratios
Lynge and Thygesen 1990	cohort	dry cleaning workers	study population overlaps with (Lynge et al. 2006), which included a larger number of individuals
Mallin 1990	cluster analysis	exposure through contaminated drinking water	extent exposure to tetrachloroethylene is unclear
Minder and Beer-Porizek 1992	cohort	laundry and dry cleaning workers	reports proportionate mortality ratios
Nakamura 1985	cohort	laundry and dry cleaning workers	reports proportionate mortality ratios
Reulen et al. 2008	meta-analysis	laundry and dry cleaning workers	meta-analysis

Sources	Study design	Exposure category	Comments
Schoenberg et al. 1984	case-control	dry cleaning workers	study population overlaps with (Smith et al. 1985) and (Silverman et al. 1989), which both include a larger number of individuals. Launderers were included in this occupational category [personal communication with Debra Silverman]
Seldén and Ahlborg 2011	cohort	laundry and dry cleaning workers	study population overlaps with (Pukkala et al. 2009), which includes a larger number of individuals
Smith et al. 1985	case-control	laundry and dry cleaning workers	study population overlaps with (Silverman et al. 1989, 1990)
Sung et al. 2007	cohort	employee of an electronics factory	extent exposure to tetrachloroethylene is unclear
Swanson and Burns 1995	case-control	dry cleaning workers	study population overlaps with (Burns and Swanson 1991), which includes a larger number of individuals
Travier et al. 2002	case-control	laundry and dry cleaning workers	study population overlaps with (Pukkala et al. 2009), which includes a larger number of individuals
Walker et al. 1997	cohort	laundry and dry cleaning workers	reports proportionate mortality ratios
Wilson et al. 2008	cohort	laundry and dry cleaning workers	study population overlaps with (Pukkala et al. 2009), which includes a larger number of individuals

References

Andersen A, Barlow L, Engeland A, Kjaerheim K, Lynge E, Pukkala E. 1999. Work-related cancer in the Nordic countries. Scand. J. Work. Environ. Health 25 Suppl 2:1–116.

Aschengrau A, Ozonoff D, Paulu C, Coogan P, Vezina R, Heeren T, et al. 1993. Cancer risk and tetrachloroethylene-contaminated drinking water in Massachusetts. Arch. Environ. Health 48:284–92.

Boice JD, Marano DE, Fryzek JP, Sadler CJ, McLaughlin JK. 1999. Mortality among aircraft manufacturing workers. Occup. Environ. Med. 56:581–97.

Burns PB, Swanson GM. 1991. Risk of urinary bladder cancer among blacks and whites: the role of cigarette use and occupation. Cancer Causes Control 2:371–9.

Chang Y-M, Tai C-F, Yang S-C, Lin RS, Sung F-C, Shih T-S, et al. 2005. Cancer incidence among workers potentially exposed to chlorinated solvents in an electronics factory. J. Occup. Health 47:171–80.

Duh RW, Asal NR. 1984. Mortality among laundry and dry cleaning workers in Oklahoma. Am. J. Public Health 74:1278–80.

Ji J, Granström C, Hemminki K. 2005. Occupation and bladder cancer: a cohort study in Sweden. Br. J. Cancer 92:1276–8.

Katz RM, Jowett D. 1981. Female laundry and dry cleaning workers in Wisconsin: a mortality analysis. Am. J. Public Health 71:305–7.

Lipworth L, Sonderman JS, Mumma MT, Tarone RE, Marano DE, Boice JD, et al. 2011. Cancer mortality among aircraft manufacturing workers: an extended follow-up. J. Occup. Environ. Med. 53:992–1007.

Lynge E, Andersen A, Rylander L, Tinnerberg H, Lindbohm M-L, Pukkala E, et al. 2006. Cancer in persons working in dry cleaning in the Nordic countries. Environ. Health Perspect. 114:213–9.

Lynge E, Thygesen L. 1990. Primary liver cancer among women in laundry and dry-cleaning work in Denmark. Scand. J. Work. Environ. Health 16:108–12.

Mallin K. 1990. Investigation of a bladder cancer cluster in northwestern Illinois. Am. J. Epidemiol. 132:S96–106.

Minder CE, Beer-Porizek V. 1992. Cancer mortality of Swiss men by occupation, 1979-1982. Scand. J. Work. Environ. Health 18 Suppl 3:1–27.

Nakamura K. 1985. Mortality patterns among cleaning workers. Sangyo Igaku. 27:24–37.

Pukkala E, Martinsen JI, Lynge E, Gunnarsdottir HK, Sparén P, Tryggvadottir L, et al. 2009. Occupation and cancer - follow-up of 15 million people in five Nordic countries. Acta Oncol. 48:646–790.

Reulen RC, Kellen E, Buntinx F, Brinkman M, Zeegers MP. 2008. A meta-analysis on the association between bladder cancer and occupation. Scand. J. Urol. Nephrol. Suppl. 64–78.

Schoenberg JB, Stemhagen A, Mogielnicki AP, Altman R, Abe T, Mason TJ. 1984. Case-control study of bladder cancer in New Jersey. I. Occupational exposures in white males. J. Natl. Cancer Inst. 72:973–81.

Seldén AI, Ahlborg G. 2011. Cancer morbidity in Swedish dry-cleaners and laundry workers: historically prospective cohort study. Int. Arch. Occup. Environ. Health 84:435–43.

Silverman DT, Levin LI, Hoover RN. 1990. Occupational risks of bladder cancer among white women in the United States. Am. J. Epidemiol. 132:453–61.

Silverman DT, Levin LI, Hoover RN. 1989. Occupational risks of bladder cancer in the United States: II Nonwhite men. J. Natl. Cancer Inst. 81:1480–3.

Smith EM, Miller ER, Woolson RF, Brown CK. 1985. Bladder cancer risk among laundry workers, dry cleaners, and others in chemically-related occupations. J. Occup. Med. 27:295–7.

Sung T-I, Chen P-C, Jyuhn-Hsiarn Lee L, Lin Y-P, Hsieh G-Y, Wang J-D. 2007. Increased standardized incidence ratio of breast cancer in female electronics workers. BMC Public Health 7:102.

Swanson GM, Burns PB. 1995. Cancer incidence among women in the workplace: a study of the association between occupation and industry and 11 cancer sites. J. Occup. Environ. Med. 37:282–7.

Travier N, Gridley G, Roos AJ De, Plato N, Moradi T, Boffetta P. 2002. Cancer incidence of dry cleaning, laundry and ironing workers in Sweden. Scand. J. Work. Environ. Health 28:341–8.

Walker JT, Burnett CA, Lalich NR, Sestito JP, Halperin WE. 1997. Cancer mortality among laundry and dry cleaning workers. Am. J. Ind. Med. 32:614–9.

Wilson RT, Donahue M, Gridley G, Adami J, Ghormli L El, Dosemeci M. 2008. Shared occupational risks for transitional cell cancer of the bladder and renal pelvis among men and women in Sweden. Am. J. Ind. Med. 51:83–99.
